# Long-Term Exposure to Ambient Air Pollution and Metabolic Syndrome in Adults

**DOI:** 10.1371/journal.pone.0130337

**Published:** 2015-06-23

**Authors:** Ikenna C. Eze, Emmanuel Schaffner, Maria Foraster, Medea Imboden, Arnold von Eckardstein, Margaret W. Gerbase, Thomas Rothe, Thierry Rochat, Nino Künzli, Christian Schindler, Nicole Probst-Hensch

**Affiliations:** 1 Swiss Tropical and Public Health Institute, Basel, Switzerland; 2 University of Basel, Basel, Switzerland; 3 Institute of Clinical Chemistry, University Hospital Zurich, Zurich, Switzerland; 4 Division of Pneumology, Geneva University Hospital, Geneva, Switzerland; 5 Zürcher Höhenklinik, Davos, Switzerland; University ofTennessee Health Science Center, UNITED STATES

## Abstract

Air pollutants (AP) play a role in subclinical inflammation, and are associated with cardiovascular morbidity and mortality. Metabolic syndrome (MetS) is inflammatory and precedes cardiovascular morbidity and type 2 diabetes. Thus, a positive association between AP and MetS may be hypothesized. We explored this association, (taking into account, pathway-specific MetS definitions), and its potential modifiers in Swiss adults. We studied 3769 participants of the Swiss Cohort Study on Air Pollution and Lung and Heart Diseases in Adults, reporting at least four-hour fasting time before venepuncture. AP exposures were 10-year mean residential PM_10_ (particulate matter <10μm in diameter) and NO_2_ (nitrogen dioxide). Outcomes included MetS defined by World Health Organization (MetS-W), International Diabetes Federation (MetS-I) and Adult Treatment Panel-III (MetS-A) using four- and eight-hour fasting time limits. We also explored associations with individual components of MetS. We applied mixed logistic regression models to explore these associations. The prevalence of MetS-W, MetS-I and MetS-A were 10%, 22% and 18% respectively. Odds of MetS-W, MetS-I and MetS-A increased by 72% (51-102%), 31% (11-54%) and 18% (4-34%) per 10μg/m^3^ increase in 10-year mean PM_10_. We observed weaker associations with NO_2_. Associations were stronger among physically-active, ever-smokers and non-diabetic participants especially with PM_10_ (p<0.05). Associations remained robust across various sensitivity analyses including ten imputations of missing observations and exclusion of diabetes cases. The observed associations between AP exposure and MetS were sensitive to MetS definitions. Regarding the MetS components, we observed strongest associations with impaired fasting glycemia, and positive but weaker associations with hypertension and waist-circumference-based obesity. Cardio-metabolic effects of AP may be majorly driven by impairment of glucose homeostasis, and to a less-strong extent, visceral adiposity. Well-designed prospective studies are needed to confirm these findings.

## Introduction

Metabolic syndrome (MetS) represents a group of symptoms including central obesity, hypertension, atherogenic dyslipidaemias and insulin resistance. World Health Organization (WHO) defines MetS (MetS-W) as diagnosis of impaired fasting glycaemia (IFG; or treatment for type 2 diabetes) and of any two out of central obesity, hypertension, hypertriglyceridemia (HTG) and low high-density lipoproteins (HDL) (or treatment for specific dyslipidaemia), and urinary albumin excretion ratio ≥20μg/min [1]. International Diabetes Federation (IDF) defines MetS (MetS-I) as central obesity and any two out of IFG, hypertension, HTG and low HDL [2], whereas Adult Treatment Panel (ATP) III defines MetS (MetS-A) as diagnosis of any three of five major components [3, 4]. MetS greatly contributes to global disease burden, occurring in about 25% of adults [2]. It predisposes to cardiovascular events and type 2 diabetes. Similarly, air pollutants (AP) are common, top risk factors for disease burden [5] and have been associated with cardiovascular [6–8]-and diabetes-related events [9–11]. Controlling disease burden from cardiovascular morbidity and diabetes implies that prevention of MetS and excessive AP exposure are crucial. Identifying modifiable risk factors to MetS will improve attribution of the burden and support public health control strategies.

MetS enhanced susceptibility to adverse effects of short-term AP exposure. Experimental exposure to diesel exhaust resulted in more haemoconcentration and thrombocytosis in MetS subjects compared to healthy ones [12]. MetS subjects also developed cardiovascular symptoms when exposed to ultrafine particles [13]. Susceptibility to low grade systemic inflammation on exposure to long- term particulate matter <10μm (PM_10_) was enhanced by MetS [14]. Thus, a link between AP exposure and MetS is plausible but has not been studied. Previous MetS-related studies have focused on PM effects. Unlike PM, which is a marker of general pollution and particle exposure, Nitrogen dioxide (NO_2_) is more specific for traffic-related pollution. Studying NO_2_ will reveal if traffic exposure contributes to the association, or whether the observed association solely reflects a particle effect (pointing towards an innate immunity activation pathway) or a contribution of different sources. Studying the various definitions of MetS will not only assess the sensitivity of associations to definition, but will also aid the understanding of pathways most likely driving the cardio-metabolic effects of AP on a population level. We therefore explored associations between long-term AP exposure and MetS in adults from a general population sample.

## Materials and Methods

### Ethics Statement

Ethical clearance for the Swiss Cohort Study on Air Pollution and Lung and Heart Diseases in Adults (SAPALDIA) was obtained from the Swiss Academy of Medical Sciences, the National Ethics Committee for Clinical Research (UREK, Project Approval Number 123/00) and the Cantonal Ethics Committees of the eight health examination areas (Aargau, Basel, Geneva, Grisons, Ticino, Valais, Vaud and Zurich). Participants were required to give written consent prior to the conduct of any health examination.

We used data from 3769 follow-up participants of the SAPALDIA study aged 29–73 years. Details of this study are explained elsewhere [15] but briefly, SAPALDIA began in 1991 with 9651 participants randomly drawn from eight Swiss communities representing a wide range of environmental conditions in Switzerland. 8047 individuals participated in the follow-up study in 2001/2002. Participants completed computer-assisted interviews on health and lifestyle, and had physical examinations including blood sampling, at follow-up, into a bio bank for biomarker and genetic assays. Inclusion in the present study required participation in the follow-up study, complete data on outcomes and covariates and at least four-hour fasting time before the follow-up examination. The reduction in sample size for this study is primarily explained by the exclusion of non-fasting subjects. Fasting status was not required for SAPALDIA participation.

### Definition of MetS

Participants reported their fasting time at first follow-up physical examination (including venepuncture). Height, weight, blood pressure (BP), plasma glucose and lipids were measured. Blood pressure was measured twice at rest, on the left arm, at least three minutes apart, in a sitting position. The mean value of both measures was computed for analyses. Participants were asked about physician diagnoses of diabetes, hypertension, dyslipidaemia and use of medication for these conditions. We defined hypertension as BP (mmHg) ≥ 140/90 (MetS-W) and >130/85 (MetS-I; MetS-A) or a physician diagnosis/ treatment. We defined low HDL as plasma HDL (mmol/l) <0.9 (MetS-W) and <1.03 (MetS-I; MetS-A) in males and <1.0 and <1.30 respectively in females and/or diagnosis/ treatment of dyslipidaemias. We defined HTG as plasma triglyceride (mmol/l) ≥1.7 and/or diagnosis/ treatment of dyslipidaemias, and impaired fasting glycaemia (IFG) as plasma glucose ≥6.1mmol/l (MetS-W) and ≥5.6mmol/l (MetS-I; MetS-A) and/or diagnosis/treatment of diabetes. Waist circumference (WC) was not measured at this visit, but was measured at the next follow-up visit. We derived a prediction model, with optimal Bayesian Information Criterion, for waist circumference measured at the next follow-up:

$$\begin{array}{l} \text{Waist circumference }\left(\text{cm}\right)\text{ = }\beta_{\text{0}}\text{+ }\beta_{\text{1}}\text{*sex+}\beta_{\text{2}}\text{*age+ }\beta_{\text{3}}{\text{*age}}^{2}\text{+ }\beta_{\text{4}}\text{*BMI+ }\beta_{\text{5}}{\text{*BMI}}^{2}\text{+}\beta_{\text{6}}\text{*age*bmi+ }\\ \beta_{\text{7}}\text{*sex*age+ }\beta_{\text{8}}{\text{*sex*age}}^{2}\text{+ }\beta_{\text{9}}\text{*sex*BMI+ }\beta_{\text{10}}{\text{*sex*BMI}}^{2}\text{+ }\beta \text{11*sex*age*BMI+ }\beta_{\text{12}}\text{*alcohol+ }\\ \beta_{\text{13}}\text{*physical activity+ }\beta_{\text{14}}\text{*ex-smoker+ }\beta_{\text{15}}\text{*current smoker.} \end{array}$$

We applied this model, using the covariate values of the second survey and added the residuals from the third survey, to back-predict waist circumference for present analyses. We used cross-validation to assess our imputation model, randomly splitting the follow-up sample into a training and a validation sample. The mean imputation error was not significantly different from zero, and the correlations of the imputation errors and the independent variables were also not significantly different from zero. The adjusted R^2^ of the imputation model was 0.79 and the squared correlation between the imputed and the actual values was of the same size.

We thus defined central obesity (MetS-I) for a European population as WC ≥94cm and ≥80cm for males and females respectively. We also defined central obesity (MetS-A) as WC ≥102cm and ≥88cm for males and females respectively. Central obesity can be assumed if BMI>30 kg/m^2^ [2]. Finally, we defined MetS-W, MetS-I and MetS-A based on the above criteria.

### Assignment of exposures

We considered estimates of residential exposure to PM_10_ and NO_2_. Annual means of AP for 1990 and 2000 were estimated from dispersion models using various emission inventories including road and rail traffic, residential, agricultural, heavy equipment and industrial emissions [16] on a 200x200m grid, and linked to participants’ addresses.[17] Estimates of NO_2_ exposure were obtained from a hybrid model incorporating land-use regression, since the dispersion model alone did not optimally predict NO_2_ near traffic sites.[18] Annual data of AP at monitoring sites and participants’ residential histories were used to estimate annual means of residential exposure levels during the follow-up period and to assign estimates of average residential exposure over the 12 month and 10 year period, respectively, preceding the follow-up examination.[17]

### Potential confounders

Consistent with our previous report on diabetes [10], we considered the following characteristics, measured at follow-up, as potential confounders: age, sex, educational attainment (≤9, >9 years), smoking status (never, former, current) and pack-years, passive smoke exposure (yes/no), occupational exposure to vapours, gases, dusts or fumes (VGDF; yes/no), alcohol consumption (including beers, wines, liquors and spirits) (never, ≤ once a day, > once a day), consumption of raw vegetables (including salads, juices), citrus fruits (including juices) and other fruits (including juices) (never, ≤ 3 days per week, >3 days per week respectively), and self-reported vigorous physical activity defined as participation in activities making one sweat or breathless (<0.5 and ≥0.5 hours/week). We also considered neighbourhood-level socio-economic index (SEI) of participants, derived from a principal component analysis using median rent, number of residents of households, educational level and occupation of household heads [19].

### Statistical Analyses

We summarized participants’ characteristics by different MetS definitions and also by inclusion/exclusion status. We estimated the prevalence of MetS-W, MetS-I and MetS-A, and their associations with 10-year-means of exposure metrics, using mixed logistic models with a random intercept for study area. Since metabolic syndrome is common [2] and given the prevalence in our study sample, we applied mixed Poisson models to estimate incidence rate ratios and used a heuristic approach to obtain robust confidence intervals [20]. Our fully-adjusted model included participants’ age, sex, educational attainment, neighbourhood SEI, smoking status and pack-years, passive smoke and VGDF exposure, consumption of alcohol, vegetables, citrus fruits and other fruits, and physical activity and BMI. We adjusted for continuous BMI to capture its variation within obesity and non-obesity groups. Using this fully-adjusted model, we also explored independent associations of PM_10_ and NO_2_ with MetS in two- pollutant models. We also explored associations between AP and components of MetS. All these models additionally included BMI except for the AP- obesity model. We repeated these analyses among participants reporting at least eight-hour fasting time (N = 367).

We assessed potential effect modification by age (≤50, >50 years), sex, and physical activity, diabetes and smoking status by stratification and interaction, given previously reports on their role as potential modifiers of AP and diabetes association [21]. Sensitivity analyses included: imputation of 75 observations (10 imputations) with missing data using chained equations; excluding participants who had IFG or obesity but not identified as MetS; treating study area as fixed factor; omitting study area from the models. We applied inverse probability weighting (IPW) to explore non-participation bias. We defined alternative MetS including MetS-I with BMI-based central obesity and MetS-I with North American cut-offs for waist circumference. We performed all analyses with STATA version 13 (Stata Corporation, Texas).

## Results

### Characteristics of participants

Table 1 shows the characteristics of included participants by MetS status. The distribution of established risk factors with MetS generally followed expectations (e.g. male sex, smoking, physical inactivity were more prevalent in MetS). The MetS cases also had higher exposures to AP than the controls (Table 1).

**Table 1:** Background Characteristics of participants.

Characteristic (%)	MetS-W^a^	MetS-I^b^	MetS-A^c^	No MetS^d^
N	382	771	663	2617
Females	40.1	46.0	40.8	58.0
Education >9 years	85.1	88.9	88.5	93.6
Never smokers	37.2	43.3	44.6	45.0
ETS exposure	49.5	46.3	46.4	46.7
Occupational exposure to VGDF	45.0	45.2	45.1	42.4
Alcohol intake: None	13.1	9.9	9.9	9.9
≤ once/day	72.2	76.4	75.3	81.7
> once/day	14.7	13.7	14.8	8.4
Citrus fruits intake: None	12.8	9.5	8.7	7.6
≤3days/week	54.2	54.5	55.7	56.8
>3days/week	33.0	36.0	35.6	35.6
Fruit intake: None	2.1	2.1	2.1	2.1
≤3days/week	26.4	30.2	30.8	33.7
>3days/week	71.5	67.7	67.1	64.2
Raw vegetables intake: None	0	1.0	0.7	0.7
≤3days/week	20.7	18.0	18.6	18.5
>3days/week	79.3	81.0	80.7	80.8
Vigorous physical activity ≥0.5hours/week	42.7	53.0	52.8	60.1
Impaired fasting glycaemia (IFG)^e^	100	56.3	67.8	7.9/20.7^h^
Low high-density lipoproteins (HDL)^f^	41.6	51.1	65.6	6.9/14.7^h^
High triglycerides	91.6	83.4	89.4	34.3
Obesity (BMI>30kg/m^2^)	49.0	36.4	34.0	9.3
Hypertension^g^	81.9	82.4	82.0	25.5/36.3^h^
Area:				
Basel	13.4	11.3	10.0	10.6
Wald	14.6	13.7	16.5	16.1
Davos	2.6	8.6	8.2	9.1
Lugano	25.1	17.6	19.8	17.3
Montana	5.2	10.1	10.5	11.6
Payerne	14.7	15.2	12.3	11.9
Aarau	16.0	14.5	13.6	13.6
Geneva	8.4	8.9	9.1	9.9
Mean (SD)				
Age (years)	61.4(7.3)	58.1 (9.1)	57.9 (9.2)	51.2 (11.5)
BMI (kg/m^2^)	30.3(4.9)	29.1(3.9)	28.7 (4.0)	24.8 (3.9)
Predicted waist circumference (cm)	100.7 (11.9)	100.3 (10.6)	98.8 (11.7)	83.5 (11.4)
Neighborhood SEI	61.7(10.3)	62.5(9.9)	62.9 (9.5)	63.2 (10.0)
Pack-years of cigarettes smoked	15.9(24.7)	13.4(22.4)	13.6 (22.2)	9.8 (16.6)
10-year PM_10_ (μg/m^3^)	25.0(7.4)	22.7(7.9)	22.8 (8.1)	22.2 (7.8)
10-year NO_2_ (μg/m^3^)	29.9(11.4)	27.6(11.6)	27.5 (11.8)	27.2 (11.3)

MetS-W: World Health Organization-defined metabolic syndrome. MetS-I: International Diabetes Federation-defined metabolic syndrome. MetS-A: Adult Treatment Panel III-defined metabolic syndrome. ETS: environmental tobacco smoke. VGDF: vapours, gases, dusts or fumes. SEI: socio-economic index expressed as a percentage. PM_10_: particulate matter <10μm in diameter from all sources. NO_2_: nitrogen dioxide.

^a^defined as IFG and any two of central obesity, hypertension, low HDL and high triglycerides.

^b^defined as central obesity and any two of IFG, hypertension, low HDL and high triglycerides.

^c^defined as any three of IFG, central obesity, hypertension, low HDL and high triglycerides.

^d^ defined as not having a, b and c.

^e^defined by WHO as fasting blood glucose≥6.1mmol/L and/or diagnosis of type2diabetes; and by IDF and ATP-III as fasting blood glucose≥5.6mmol/L and/or diagnosis of type2diabetes. High triglycerides defined as fasting triglycerides≥1.7mmol/L or treatment for this condition.

^f^ defined by WHO as ≤ 0.9 mmol/L (males), ≤ 1.0 mmol/L (females); and by IDF and ATP-III as < 1.03 mmol/L (males), < 1.29 mmol/L (females), or treatment for this condition.

^g^defined by WHO as ≥140/90, or treatment of previously diagnosed hypertension; and by IDF and ATP-III as blood pressure >130/85 mm Hg or previously diagnosed hypertension.

^h^proportion in controls according to MetS-W/ MetS-I or MetS-A criteria respectively.

MetS-W had a weakly positive correlation with MetS-I (kappa = 0.25), but both correlated better with the MetS-A (kappa = 0.40 and 0.67 respectively). Differences between included and excluded participants are shown in S1 Table. Included participants tended to be older, more educated, never-smokers, more exposed to occupational dusts and less physically active (S1 Table).

### Associations between AP and MetS

The odds of MetS-W, MetS-I and MetS-A increased by 72% (46–102%), 31% (11–54%) and 18% (4–34%) per 10μg/m^3^ increase in 10-year mean home outdoor PM_10_ (Table 2). We also observed positive but less strong associations per 10μg/m^3^ increase in 10-year mean home outdoor NO_2_ (Table 2).

**Table 2:** Association between air pollutants and metabolic syndrome (4-hour fasting time).

	Model	10-year mean PM_10_	P-Value	10-year mean NO_2_	P-value
		OR (95%CI)		OR (95%CI)	
MetS-W	Model 1	1.64 (1.35, 1.98)	<0.001	1.20 (1.02, 1.41)	0.025^c^
Cases = 382	Model 2	1.58 (1.29, 1.95)	<0.001	1.21 (1.02, 1.43)	0.026^c^
	Model 3	1.72 (1.46, 2.02)	<0.001	1.22 (1.02, 1.46)	0.033^c^
MetS-I^a^	Model 1	1.23 (1.05, 1.45)	0.009	1.10 (1.00, 1.22)	0.056
Cases = 771	Model 2	1.21 (0.99, 1.49)	0.058	1.10 (0.97, 1.24)	0.154
	Model 3	1.31 (1.11, 1.54)	0.002	1.17 (1.04, 1.31)	0.011
MetS-A^b^	Model 1	1.12 (1.00, 1.24)	0.047^c^	1.03 (0.95, 1.10)	0.505
Cases = 663	Model 2	1.10 (0.98, 1.24)	0.117	1.01 (0.93, 1.09)	0.899
	Model 3	1.18 (1.04, 1.34)	0.011	1.05 (0.95, 1.17)	0.339

MetS-W: World Health Organization-defined metabolic syndrome. MetS-I: International Diabetes Federation-defined metabolic syndrome. Model 1: Crude; Model 2: Model 1+ age, sex, educational attainment, neighborhood socio-economic index, occupational exposure to vapors, gases, dusts or fumes, smoking status, smoked pack-years, exposure to passive smoke, consumption of fruits and raw vegetables, and physical activity; Model 3: Model 2+ body mass index. PM_10_: particulate matter <10μm in diameter from all sources. NO_2_: nitrogen dioxide. OR: odds ratio. CI: confidence interval. OR values refer to increments of 10μg/m^3^ in PM_10_ and NO_2_ exposure respectively. Participants’ study area was treated as a random effect in all models.

^a^ MetS-I defined using predicted waist circumference and European cut-off for central obesity (≥94cm for men and ≥80cm for women).

^b^ MetS-A defined using predicted waist circumference and North-American cut-off for central obesity (≥102cm for men and ≥88cm for women).

^C^ Lost statistical significance following Bonferroni correction at P<0.016 (0.05/3). PM_10_ and NO_2_ are not testing independent hypothesis.

Translated into incidence rate ratios, the risk of MetS-W, MetS-I and MetS-A increased by 52% (35–70%), 12% (4–19%), and 9% (0–19%) per 10μg/m^3^ increase in 10-year mean PM_10_, and weaker associations were also observed with NO_2_ (S2 Table). Among the outcomes, we observed strongest associations with MetS-W, and associations were stronger with PM_10_ than NO_2_ (Table 2). Restriction of analyses to subjects reporting eight-hour fasting time provided similar results albeit with limited statistical power. While odds ratios for MetS-W slightly decreased, those for MetS-I and MetS-A increased, and no association was observed between NO_2_ and MetS-A (S3 Table). In multi-pollutant MetS models, associations with PM_10_ persisted across outcomes, while those with NO_2_ were strongly decreased or lost (S4 Table).

### Modification of AP and MetS association

**Fig 1:**
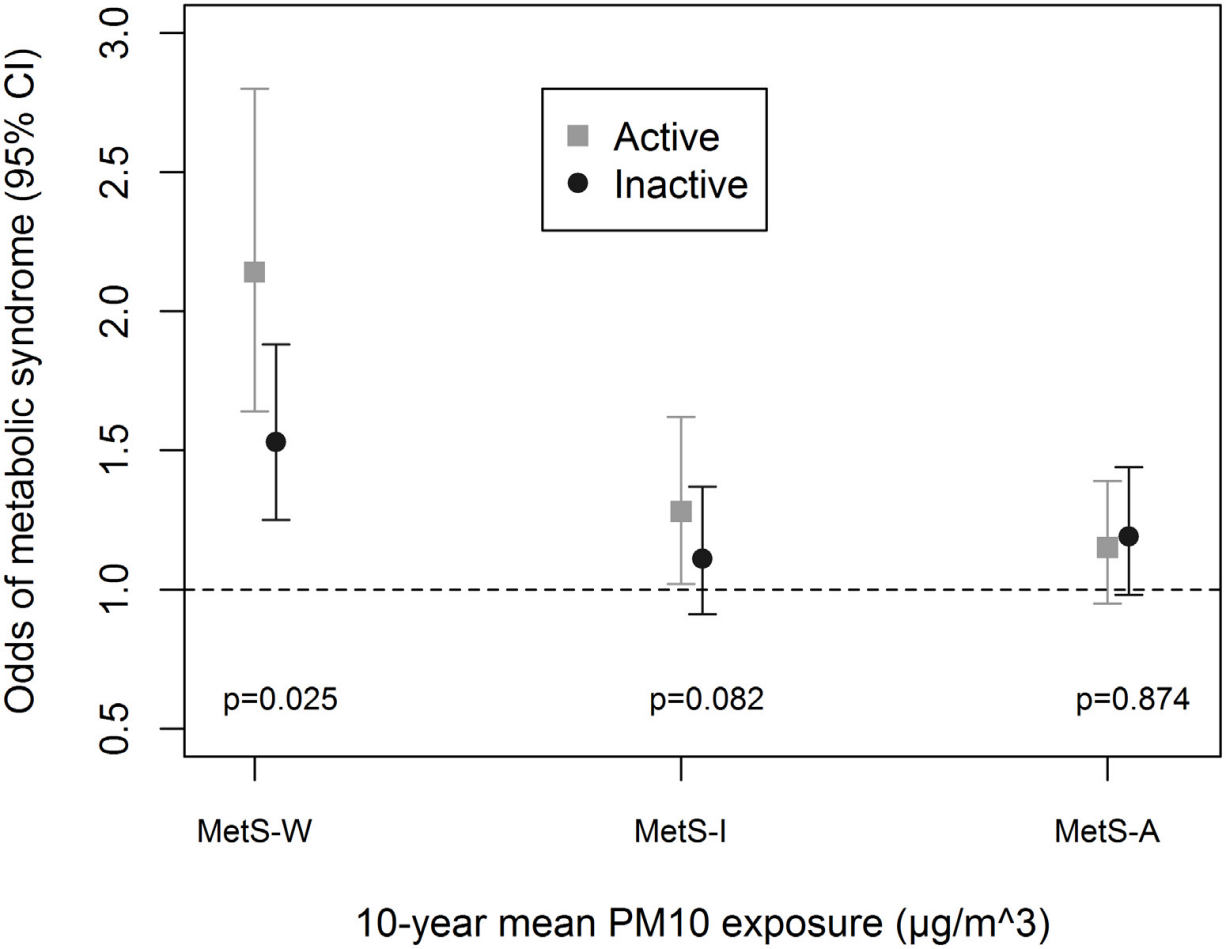
Effect modification by vigorous physical activity. MetS-W: Metabolic syndrome according to World Health Organization. MetS-I: Metabolic syndrome according to International Diabetes Federation. MetS-A: Metabolic syndrome according to Adult Treatment Panel-III criteria. Active defined as vigorous physical activity ≥30 minutes per week. Inactive defined as vigorous physical activity <30minutes per week. Fully adjusted models include age, sex, educational attainment, neighbourhood socio-economic index, occupational exposure to vapours, gases, dusts and fumes, smoking status, smoked pack-years, exposure to passive smoke, consumption of fruits and raw vegetables, and body mass index. PM_10_: particulate matter <10μm in diameter from all sources. All analyses were done with four-hour fasting participants. Participants’ study area was treated as a random effect in all models. Odds ratio values refer to increments of 10μg/m^3^ in PM_10_ exposure. Total N = 3684; N(physically-active) = 2115.

**Fig 2:**
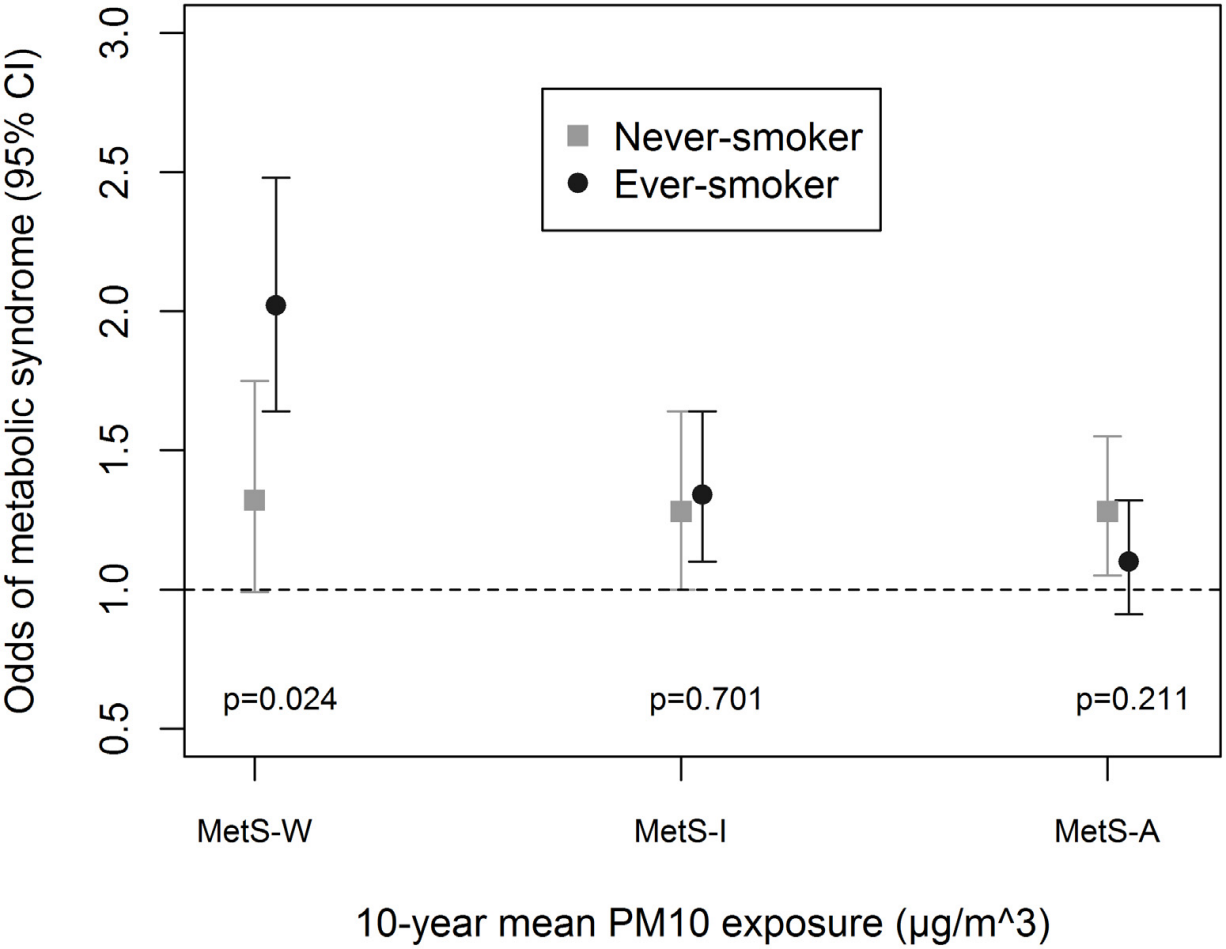
Effect modification by smoking status. MetS-W: Metabolic syndrome according to World Health Organization. MetS-I: Metabolic syndrome according to International Diabetes Federation. MetS-A: Metabolic syndrome according to Adult Treatment Panel-III criteria. Fully adjusted models include age, sex, educational attainment, neighbourhood socio-economic index, occupational exposure to vapours, gases, dusts and fumes, exposure to passive smoke, consumption of fruits and raw vegetables, physical activity and body mass index. PM_10_: particulate matter <10μm in diameter from all sources. All analyses were done with four-hour fasting participants. Participants’ study area was treated as a random effect in all models. Odds ratio values refer to increments of 10μg/m^3^ in PM_10_ exposure. Total N = 3684; N(never-smoker) = 1623.

**Fig 3:**
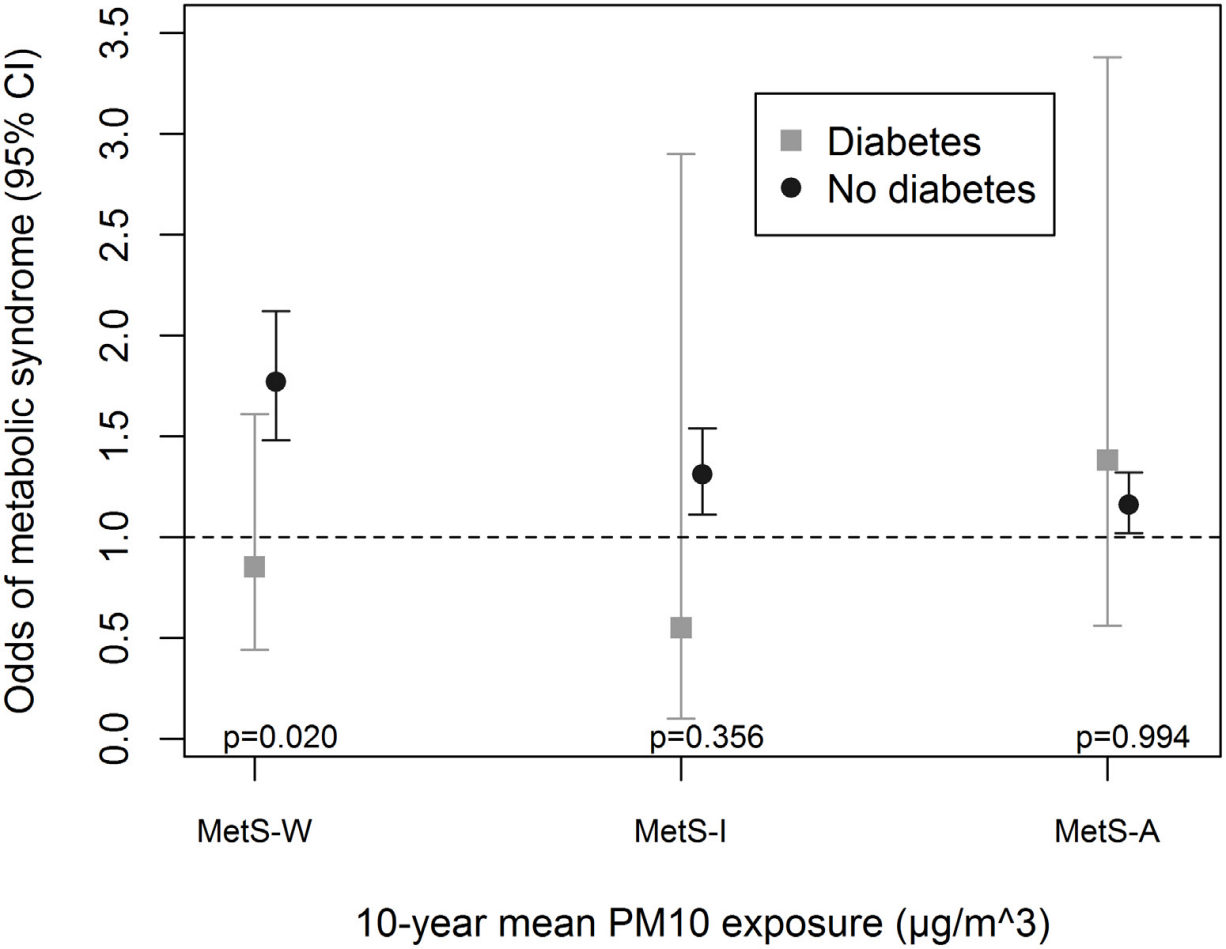
Effect modification by diabetes status. MetS-W: Metabolic syndrome according to World Health Organization. MetS-I: Metabolic syndrome according to International Diabetes Federation. MetS-A: Metabolic syndrome according to Adult Treatment Panel-III criteria. Fully adjusted models include age, sex, educational attainment, neighbourhood socio-economic index, occupational exposure to vapours, gases, dusts and fumes, smoking status, smoked pack-years, exposure to passive smoke, consumption of fruits and raw vegetables, physical activity and body mass index. PM_10_: particulate matter <10μm in diameter from all sources. All analyses were done with four-hour fasting participants. Participants’ study area was treated as a random effect in all models. Odds ratio values refer to increments of 10μg/m^3^ in PM_10_ exposure. Total N = 3684; N(diabetes) = 144.

**Fig 4:**
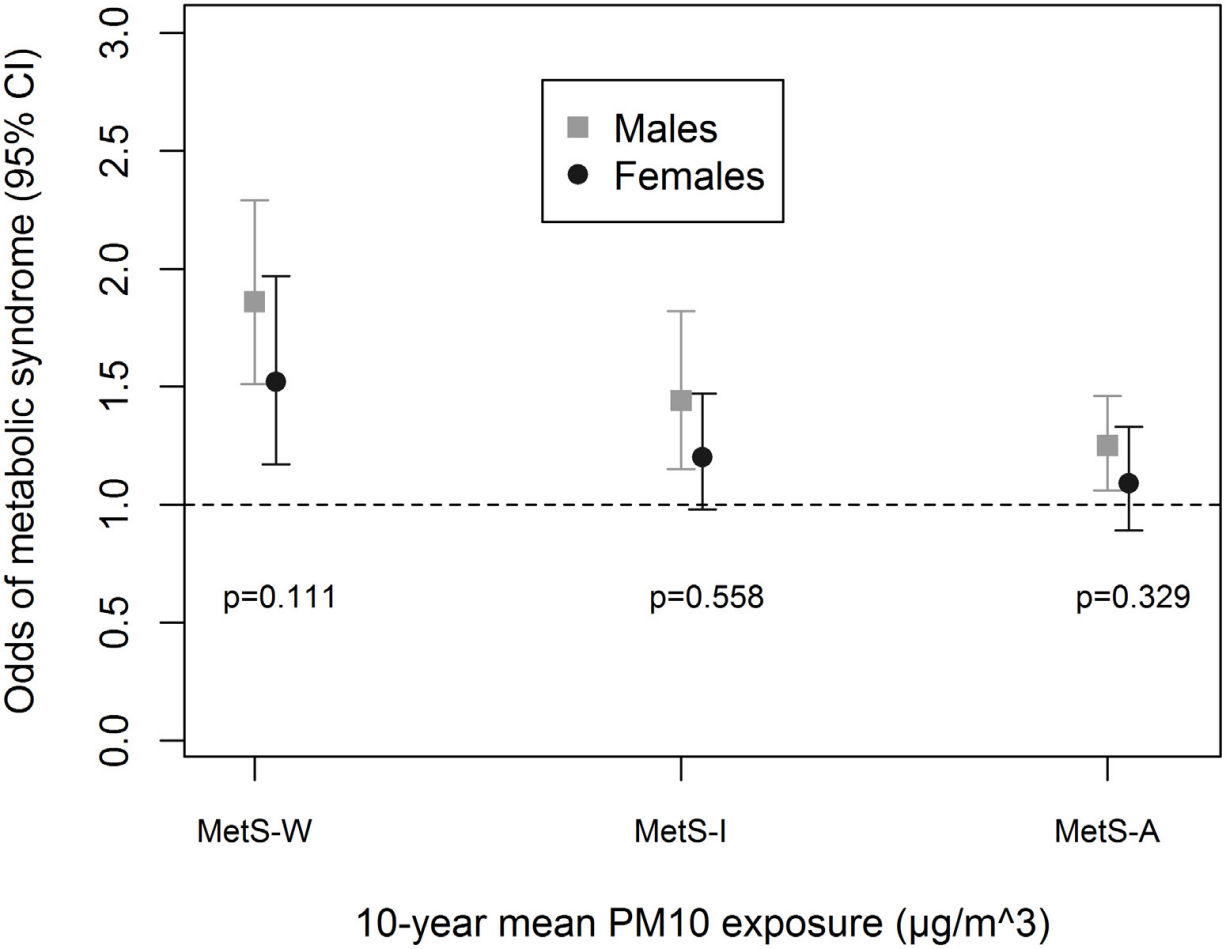
Effect modification by sex. MetS-W: Metabolic syndrome according to World Health Organization. MetS-I: Metabolic syndrome according to International Diabetes Federation. MetS-A: Metabolic syndrome according to Adult Treatment Panel-III criteria. Fully adjusted models include age, educational attainment, neighbourhood socio-economic index, occupational exposure to vapours, gases, dusts and fumes, smoking status, smoked pack-years, exposure to passive smoke, consumption of fruits and raw vegetables, physical activity and body mass index. PM_10_: particulate matter <10μm in diameter from all sources. NO_2_: nitrogen dioxide. All analyses were done with four-hour fasting participants. Participants’ study area was treated as a random effect in all models. Odds ratio values refer to increments of 10μg/m^3^ in PM_10_ exposure. Total N = 3684; N(males) = 1746.

**Fig 5:**
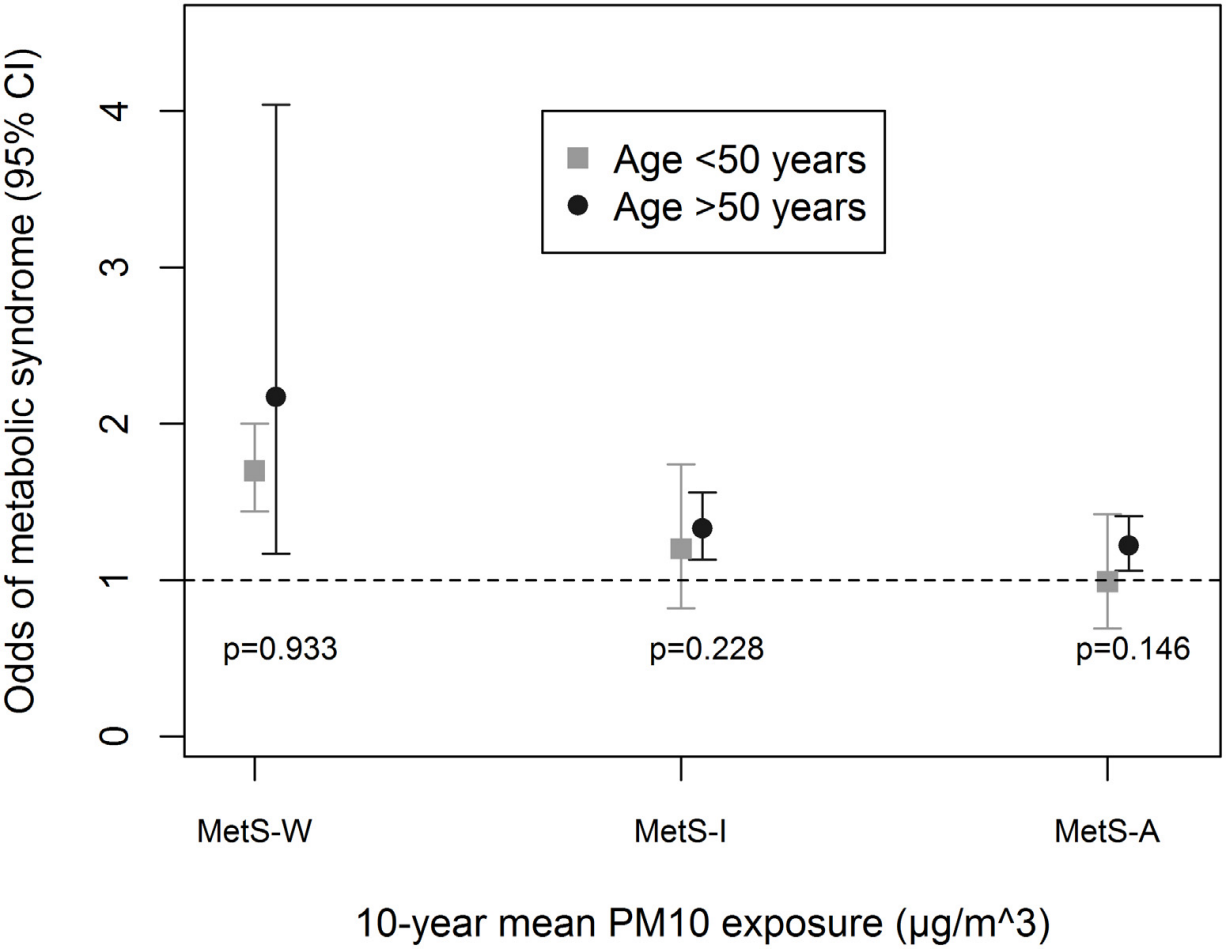
Effect modification by age group. MetS-W: Metabolic syndrome according to World Health Organization. MetS-I: Metabolic syndrome according to International Diabetes Federation. MetS-A: Metabolic syndrome according to Adult Treatment Panel-III criteria. Fully adjusted models include sex, educational attainment, neighbourhood socio-economic index, occupational exposure to vapours, gases, dusts and fumes, smoking status, smoked pack-years, exposure to passive smoke, consumption of fruits and raw vegetables, physical activity and body mass index. PM_10_: particulate matter <10μm in diameter from all sources. All analyses were done with four-hour fasting participants. Participants’ study area was treated as a random effect in all models. Odds ratio values refer to increments of 10μg/m^3^ in PM_10_ exposure. Total N = 3684; N(age≤50) = 1393.

Associations were enhanced by being physically-active (Fig 1), an ever-smoker (Fig 2) and non-diabetic (Fig 3). We observed significant interaction between these variables and PM_10_ in association with MetS-W (P_interaction_ = 0.025, 0.024 and 0.020 respectively). Similar trends were observed with MetS-I, and associations with NO_2_ even though interaction terms were non-significant (S5 Table). We observed no significant gender (Fig 4 and S5 Table) and age-group (Fig 5 and S5 Table) differences in the AP-MetS association, even though there was indication for a stronger association among males and participants >50 years (Figs 4 and 5, S5 Table). With MetS-A, there was a significant modification of NO_2_ effect by age (P_interaction_ = 0.021; S5 Table). Other interactions were largely non-significant (S5 Table).

### Sensitivity Analyses

Estimates of associations were remarkably robust across sensitivity analyses. Multiple imputations of 75 observations marginally improved effect estimates. IPW adjustment for participation bias and exclusion of diabetes cases did not appreciably change these estimates (Table 3). Ignoring study area gave very similar results as the fully-adjusted random-effects model whereas area-specific slopes reduced the effect estimates especially for PM_10_ (Table 3).

**Table 3:** Sensitivity Analyses.

	10-year mean PM_10_			10-year mean NO_2_		
	MetS-W	MetS-I	MetS-A	MetS-W	MetS-I	MetS-A
	OR (95%CI)	OR (95%CI)	OR (95%CI)	OR (95%CI)	OR (95%CI)	OR (95%CI)
Fully-adjusted, random-effect model	1.72 (1.46, 2.02)	1.31 (1.11, 1.54)	1.18 (1.04, 1.34)	1.22 (1.02, 1.46)	1.17 (1.04, 1.31)	1.05 (0.95, 1.17)
P-value	<0.001	0.002	0.011	0.033	0.011	0.339
Fully-adjusted random-effect model with multiple imputations	1.81 (1.52, 2.15)	1.39 (1.20, 1.62)	1.17 (1.02,1.35)	1.28 (1.15, 1.43)	1.23 (1.11, 1.12)	1.07 (0.98, 1.17)
P-value	<0.001	<0.001	0.021	<0.001	<0.001	0.156
IPW analysis for participation bias.	1.74 (1.49, 2.03)	1.29 (1.12, 1.49)	1.17 (1.02, 1.33)	1.31 (1.19, 1.46)	1.15 (1.04, 1.27)	1.05 (0.96, 1.15)
P-value	<0.001	0.001	0.023	<0.001	0.005	0.292
Model excluding diabetes cases	1.77 (1.48, 2.12)	1.31 (1.11, 1.54)	1.16 (1.02, 1.32)	1.22 (1.00, 1.50)	1.17 (1.05, 1.32)	1.04 (0.94, 1.16)
P-value	0.020	0.356	0.994	0.110	0.091	0.597
Model excluding diabetes cases reporting medication	1.80 (1.51, 2.14)	1.30 (1.10, 1.53)	1.17 (1.03, 1.34)	1.15 (0.92, 1.43)	1.17 (1.04, 1.32)	1.05 (0.94, 1.16)
P-value	<0.001	0.002	0.015	0.226	0.009	0.421
Model, ignoring study area	1.72 (1.46, 2.02)	1.30 (1.13, 1.50)	1.18 (1.04, 1.34)	1.31 (1.18, 1.46)	1.16 (1.06, 1.28)	1.06 (0.98, 1.16)
P-value	<0.001	<0.001	0.011	<0.001	0.002	0.159
Model, including study area as fixed effect	1.10 (0.63, 2.09)	1.35 (0.86, 2.11)	1.19 (0.74, 1.91)	1.09 (0.88, 1.36)	1.21 (0.99, 1.48)	0.96 (0.79, 1.14)
P-value	0.733	0.194	0.474	0.419	0.058	0.576

Fully adjusted models include age, sex, educational attainment, neighbourhood socio-economic index, occupational exposure to vapours, gases, dusts and fumes, smoking status, smoked pack-years, exposure to passive smoke, consumption of fruits and raw vegetables, physical activity and body mass index. MI: multiple imputations. IPW: inverse probability weighting. PM_10_: particulate matter <10μm in diameter from all sources. NO_2_: nitrogen dioxide. OR: odds ratio. CI: confidence interval. OR refer to increments of 10μg/m^3^ in PM_10_, and NO_2_ exposure respectively. All analyses were done with four-hour fasting participants.

We observed weaker associations with MetS-I based on BMI-defined central obesity, and MetS-I based on North-American cut-offs for central obesity in a European population (S6 Table).

### Association between AP and MetS components

There were positive associations between AP and IFG (Table 4). Associations were consistent across exposure metrics. We also observed positive associations with hypertension, which were strongest with NO_2_. We also found stronger associations with central obesity defined by waist circumference compared to central obesity defined by BMI. We found no appreciable associations with other components, although eight-hour MetS estimates appeared to be stronger than four-hour MetS estimates (Table 4).

**Table 4:** Association between air pollutants and components of metabolic syndrome.

	Fasting time (hours)	10-year mean PM_10_ OR (95%CI)	P-value	10-year mean NO_2_ OR (95%CI)	P-value
Impaired fasting Glycaemia (IFG;WHO)	4	1.82 (1.60, 2.08)	<0.001	1.15 (0.98, 1.34)	0.080
	8	2.27 (1.43, 3.62)	0.001	1.33 (0.98, 1.79)	0.063
Impaired fasting Glycaemia (IFG; IDF/ATP-III)	4	1.45 (1.19, 1.78)	<0.001	1.06 (0.93, 1.21)	0.388
	8	1.84 (1.30, 2.60)	0.001	1.36 (1.08, 1.72)	0.008
Low high-density lipoproteins (WHO)	4	0.95 (0.76, 1.19)	0.657	0.88 (0.76, 1.01)	0.071
	8	0.89 (0.47, 1.70)	0.735	0.76 (0.49, 1.19)	0.229
Low high-density lipoproteins (IDF/ATP-III)	4	0.99 (0.87, 1.12)	0.847	0.95 (0.87, 1.05)	0.303
	8	0.99 (0.63, 1.56)	0.982	0.86 (0.66, 1.13)	0.287
High triglycerides	4	0.90 (0.77, 1.05)	0.169	0.94 (0.85, 1.03)	0.194
	8	1.14 (0.78, 1.67)	0.494	0.94 (0.73, 1.21)	0.630
Hypertension (WHO)	4	1.12 (0.97, 1.29)	0.130	1.11 (1.01, 1.20)	0.022
Hypertension (IDF/ATP-III)	4	1.11 (0.95, 1.30)	0.172	1.12 (1.03, 1.23)	0.011
Central obesity (BMI>30kg/m2)	4	1.00 (0.83, 1.21)	0.971	1.01 (0.89, 1.14)	0.898
Central obesity^a^	4	1.19 (0.90, 1.58)	0.218	1.06 (0.90, 1.26)	0.465

Fully adjusted models include age, sex, educational attainment, neighbourhood socio-economic index, occupational exposure to vapours, gases, dusts and fumes, smoking status, smoked pack-years, exposure to passive smoke, consumption of fruits and raw vegetables, physical activity and body mass index (BMI). Model for obesity excludes BMI. PM_10_: particulate matter <10μm in diameter from all sources. NO_2_: nitrogen dioxide. Traffic PM_10_ refers to dispersion models including only traffic-related emissions. OR: odds ratio. CI: confidence interval. OR values represent fold increase in odds of outcomes per 10μg/m^3^ of PM_10_, NO_2_, and 1μg/m^3^ of traffic PM_10_ exposure. IFG defined as fasting blood glucose≥6.1mmol/L and/or diagnosis of type2diabetes. High triglycerides defined as fasting triglycerides≥1.7mmol/L or treatment for this condition. Low HDL defined by IDF and ATP-III as < 1.03 mmol/L (males), < 1.29 mmol/L (females), or treatment for this condition, and by WHO as ≤ 0.9 mmol/L (males), ≤ 1.0 mmol/L (females). Hypertension defined by IDF and ATP-III as blood pressure >130/85 mm Hg and by WHO as ≥140/90, or treatment of previously diagnosed hypertension. Participants’ study area was treated as a random effect in all models. N (4 hours fasting time) = 3684. N (8 hours fasting time) = 367.

^a^Central obesity defined using the predicted waist circumference and European cut-offs (≥94cm for males and ≥80cm for females)

## Discussion

We found positive associations between markers of long-term AP exposure and MetS, which were sensitive to definition in this sample of Swiss adults. Associations were most pronounced with MetS-W, which reflects a glucose metabolism-dependent pathway, and weaker with MetS-I which is based on visceral adiposity, and MetS-A which does not depend on a particular pathway. Our results therefore suggest that AP seems to impact particularly on insulin resistance part of MetS—aligned with impact on adipose tissue inflammation observed in animal models [22–24] and homeostatic model of insulin resistance observed in humans [25, 26]. Given the cross-sectional nature of the analysis and the sub-group findings, one cannot derive etiologic conclusions. But the plausibility of underlying mechanisms warrants further longitudinal investigations of these highly relevant results.

### Potential mechanisms of action

MetS reflects a status of low grade systemic inflammation, and exposure to PM has been associated with blood markers of inflammation [27]. Exposure to PM_10_ increased the expression of inflammatory and MetS genes in mice [28]. MetS may predispose to the expression of inflammatory markers [14] and autonomic dysfunction [22, 23, 29] associated with chronic AP exposure. The components of MetS have also been positively linked to AP. Exposure to AP has been linked to hypertension [30, 31], alterations in blood lipids [32, 33], insulin resistance [22, 23] and obesity [34, 35]. Exposure to passive smoke, a contributor to PM also induces inflammatory responses and lipid changes, and has been positively associated with MetS-I [36]. In addition, sub-acute exposures to low levels of PM_2.5_ induced insulin resistance in healthy young adults [26], whereas exposure to ambient levels of PM_10_ and NO_2_ induced insulin resistance in children [25]. Based on the evidence from human insulin resistance studies, our finding of strongest association with MetS-W and the results of the individual MetS components, the insulin resistance pathway may be the strongest pathway through which AP exert their cardio-metabolic effects. This is also supported by the finding of slightly stronger association with waist circumference-based central obesity as opposed to BMI-based central obesity, with the former being a better indicator for insulin resistance.

Changes in inflammatory markers and blood lipids were non-significant in young adults when exposed to AP [37]. Conversely, significant changes were observed in middle-aged/older subjects, reversible with omega-3-fatty acid [38]. This supports our finding of stronger associations among older people. Smoking is a known risk factor for cardio-metabolic diseases [39], hence our finding of stronger effect among ever-smokers may be additive effect on the already existing effect of smoking exposure. Stronger effects among ever-smokers was observed for MetS-W and MetS-I, but not for MetS-A. This may be explained by the facts that the never-smokers, in our study population, were less physically-active (S7 Table) and had higher predicted waist circumference (90 vs. 88cm) compared to ever-smokers. Whereas the findings for MetS-W and MetS-I appear to contradict a previous finding of stronger AP effects (on diabetes) among never-smokers [21], our finding with MetS-A supports it. We did not observe any associations among the diabetes cases. This may be because of their use of medication for blood glucose control. It may also be due their very small number which limits the statistical power to see any associations.

We observed stronger associations among the physically active. This observation was independent of MetS definition and persisted in the sub-sample with eight-hour fasting time. Stronger AP associations among the physically active (with diabetes) were shown elsewhere [10, 21]. This may be expected if the physically-active spend more time outdoors, thus, their outdoor concentrations may better capture their actual exposure. Also, due to their deeper inhalation while active, the physically-active have higher exposure of their lung tissues to AP for the same ambient concentration. Physical activity improves lung function [40] and has been shown to enhance response to volatile organic compounds [41].

As shown (S7 Table), the physically-active lived in less polluted areas. Being physically inactive was also associated with areas of high outdoor PM_2.5_ concentrations in normal-weight people in previous studies [42]. One may conjecture that the observed interaction with physical activity may be partly due to some other non-considered covariates. The inactive subjects were exposed to other risk factors for MetS at a higher level than the active subjects (S7 Table), thus, the relative role of AP in MetS development may be less crucial in them. Use of more objective measures of visceral adiposity should improve the definition of MetS.

### Strengths and Limitations

This study derives from the large SAPALDIA database, with detailed information on health, socio-demographic and lifestyle characteristics. This allowed us to have a clean case definition and detailed confounder adjustment. We had validated annual estimates of residential AP exposures from which long-term exposure estimates were derived. To the best of our knowledge, this is the first study to assess direct associations between AP and MetS. Its results may help in understanding the pathways involved in the effects of AP on cardiovascular disease and diabetes.

A major limitation is the cross-sectional design which precludes etiologic inferences. We did not measure waist circumference at this visit but had a validated prediction model based on trends at the next follow-up visit. As we do not have urinary albumin excretion ratio for our participants, we may have misclassified some MetS-W cases. We used four-hour fasting time to define MetS, instead of conventional eight hours in our main analysis. This was due to the small sample of participants who reported a fasting time of at least eight hours, limiting our statistical power. However, associations were also positive in the subjects who fasted for eight hours. Four-hour fasting blood samples can be used for patient diagnosis in ambulatory settings [43]. Also, non-fasting triglycerides were shown to be a predictor of cardiac events in women [44]. PM_2.5_ was not modelled in this study, thus we relied on PM_10_. While one may argue PM_2.5_ to be more relevant for systemic effects, the lack thereof, is unlikely to bias this analysis. In Switzerland, PM_2.5_ contributes 70–80% to the PM_10_ fraction and both are highly correlated within and across SAPALDIA areas (R~0.8).

We used two markers of ambient pollution with partly different characteristics. Our results indicate possible larger effects of PM_10_ compared to NO_2_. This may largely be because PM_10_ represents a mixture of different particles, unlike NO_2_ which measures a specific gas. Particulate matter has been shown to be stronger activators of innate immunity in comparison with gaseous pollutants [22, 23].

We did not have estimates of indoor or occupational AP for our participants, but any misclassification that could be caused by this is expected to be non-systematic, leading to a null bias. We considered occupational exposure to VGDF, which partly adjusts for indoor occupational exposure. Only 46% of follow-up and 38% of baseline participants was studied. A substantial percentage of non-inclusion was due to subjects who had venepuncture in less than four-hour fasting time. Despite this low participation, all study areas and other characteristics were well represented in this study sample. Sensitivity analyses using IPW suggested that participation bias was non-substantial. Despite this finding, some bias may still persist. The weaker precision from the fixed effect model, especially for PM_10_, could be due to poor within-area spatial contrasts exhibited by PM_10_ compared to the traffic-related exposures [10, 45].

It is unclear if the associations with PM_10_ are due to the inflammation elicited by physical effects of particles and/or the innate immunity response elicited by its biological components. These and other questions deserve further investigation by future well-designed longitudinal studies. The studies should consider measured waist circumference as a component of MetS, and explore associations with PM components. Also, physical activity must be more objectively measured. Our findings, if confirmed, are of great public health relevance, as they may call for physical activity promotion to be adapted to various environmental contrasts.

## Supporting Information

S1 Table Characteristics of participants included and excluded in the study. ETS: environmental tobacco smoke. VGDF: vapours, gases, dusts and fumes. MVPA: moderate to vigorous physical activity. Hypertension defined as blood pressure >130/85 mm Hg or treatment of previously diagnosed hypertension. SEI: socio-economic index expressed as a percentage. PM_10_: particulate matter <10μm in diameter from all sources. NO_2_: nitrogen dioxide.
(DOCX)

S2 Table Incidence rate ratios of metabolic syndrome in association with air pollutants. MetS-W: World Health Organization-defined metabolic syndrome. MetS-I: International Diabetes Federation-defined metabolic syndrome. Model 1: Crude; Model 2: Model 1+ age, sex, educational attainment, neighbourhood socio-economic index, occupational exposure to vapours, gases, dusts or fumes, smoking status, smoked pack-years, exposure to passive smoke, consumption of fruits and raw vegetables, and physical activity; Model 3: Model 2+ body mass index. PM_10_: particulate matter <10μm in diameter from all sources. NO_2_: nitrogen dioxide. OR: odds ratio. CI: confidence interval. OR values refer to increments of 10μg/m^3^ in PM_10_ and NO_2_ exposure respectively. Participants’ study area was treated as a random effect in all models. N = 3684
(DOCX)

S3 Table Association between air pollutants and metabolic syndrome (8 hours fasting time). MetS-W: World Health Organization-defined metabolic syndrome. MetS-I: International Diabetes Federation-defined metabolic syndrome. Model 1: Crude; Model 2: Model 1+ age, sex, educational attainment, neighbourhood socio-economic index, occupational exposure to vapours, gases, dusts or fumes, smoking status, smoked pack-years, exposure to passive smoke, consumption of fruits and raw vegetables, and physical activity; Model 3: Model 2+ body mass index. PM_10_: particulate matter <10μm in diameter from all sources. NO_2_: nitrogen dioxide. OR: odds ratio. CI: confidence interval. OR values refer to increments of 10μg/m^3^ in PM_10_ and NO_2_ exposure respectively. Participants’ study area was treated as a random effect in all models. N = 367
(DOCX)

S4 Table Association between air pollutants and metabolic syndrome (two-pollutant models). MetS-W: World Health Organization-defined metabolic syndrome. MetS-I: International Diabetes Federation-defined metabolic syndrome. Model 1: Crude; Model 2: Model 1+ age, sex, educational attainment, neighbourhood socio-economic index, occupational exposure to vapours, gases, dusts or fumes, smoking status, smoked pack-years, exposure to passive smoke, consumption of fruits and raw vegetables, and physical activity; Model 3: Model 2+ body mass index. PM_10_: particulate matter <10μm in diameter from all sources. NO_2_: nitrogen dioxide. OR: odds ratio. CI: confidence interval. OR values refer to increments of 10μg/m^3^ in PM_10_ and NO_2_ exposure respectively. Participants’ study area was treated as a random effect in all models. N = 3684
(DOCX)

S5 Table Effect modification of NO_2_ and metabolic syndrome association. Fully adjusted models include age, sex, educational attainment, neighbourhood socio-economic index, occupational exposure to vapours, gases, dusts and fumes, smoking status, smoked pack-years, exposure to passive smoke, consumption of fruits and raw vegetables, physical activity and body mass index. NO_2_: nitrogen dioxide. All analyses were done with four-hour fasting participants. Participants’ study area was treated as a random effect in all models. OR: Odds ratios OR values refer to increments of 10μg/m^3^ in NO_2_ exposure. Total N = 3684; N(age≤50) = ; N(males) = 1746; N(physically-active) = 2115; N(never-smoker) = 1623; N(diabetes) = 144.
(DOCX)

S6 Table Association between air pollutants and alternative definitions of metabolic syndrome. MetS-W: World Health Organization-defined metabolic syndrome. MetS-I: International Diabetes Federation-defined metabolic syndrome. MetS-ATP-III: Adult treatment panel III criteria- defined metabolic syndrome. Model 1: Crude; Model 2: Model 1+ age, sex, educational attainment, neighbourhood socio-economic index, occupational exposure to vapours, gases, dusts or fumes, smoking status, smoked pack-years, exposure to passive smoke, consumption of fruits and raw vegetables, and physical activity; Model 3: Model 2+ body mass index. PM_10_: particulate matter <10μm in diameter from all sources. NO_2_: nitrogen dioxide. OR: odds ratio. CI: confidence interval. OR values refer to increments of 10μg/m^3^ in PM_10_ and NO_2_ exposure respectively. Participants’ study area was treated as a random effect in all models. N (Four-hour fasting time) = 3684; N (Eight-hour fasting time) = 367.
(DOCX)

S7 Table Participants’ characteristics by self-reported physical activity. MetS-W: Metabolic syndrome according to World Health Organization. MetS-I: Metabolic syndrome according to International Diabetes Federation. MetS-A: Metabolic syndrome according to Adult Treatment Panel III criteria. ETS: environmental tobacco smoke. VGDF: vapours, gases, dusts and fumes. IFG defined as fasting blood glucose≥6.1mmol/L and/or diagnosis of type2diabetes. High triglycerides defined as fasting triglycerides≥1.7mmol/L or treatment for this condition. Low HDL defined by IDF as < 1.03 mmol/L (males), < 1.29 mmol/L (females), or treatment for this condition, and by WHO as ≤ 0.9 mmol/L (males), ≤ 1.0 mmol/L (females). Hypertension defined by IDF and ATP-III as blood pressure >130/85 mm Hg and by WHO as ≥140/90, or treatment of previously diagnosed hypertension. SEI: socio-economic index. PM_10_: particulate matter <10μm in diameter from all sources. NO_2_: nitrogen dioxide.
(DOCX)
