# Supplementary material for: Long-Term Exposure to Ambient Air Pollution and Metabolic Syndrome in Adults
Source: PLoS One. 2015 Jun 23;10(6):e0130337. doi: 10.1371/journal.pone.0130337 (PMC4478007; doi:10.1371/journal.pone.0130337)
Supplement: S1 Table — ETS: environmental tobacco smoke. VGDF: vapours, gases, dusts and fumes. MVPA: moderate to vigorous physical activity. Hypertension defined as blood pressure >130/85 mm Hg or treatment of previously diagnosed hypertension. SEI: socio-economic index expressed as a percentage. PM10: particulate matter <10μm in diameter from all sources. NO2: nitrogen dioxide. (DOCX) [file pone.0130337.s001.docx]

S1 Table: Characteristics of participants included and excluded in the study

| Characteristic (%) | Included  (N=3684) | Excluded  (N=5967) | P-value  (Chi^2^) |
| --- | --- | --- | --- |
| Females | 52.6 | 51.1 | 0.223 |
| Education >9 years | 92.0 | 61.0 | <0.001 |
| Smoking status: Never | 44.0 | 40.6 | <0.001 |
| Former | 32.0 | 31.2 |  |
| Current | 24.0 | 28.2 |  |
| ETS exposure | 46.8 | 48.0 | 0.256 |
| Occupational exposure to VGDF | 43.0 | 19.5 | <0.001 |
| Alcohol intake: None | 10.1 | 8.0 | <0.001 |
| ≤ once/day | 79.9 | 83.9 |  |
| > once/day | 9.9 | 8.1 |  |
| Citrus fruits intake: None | 8.4 | 8.3 | 0.989 |
| ≤3days/week | 56.0 | 56.1 |  |
| >3days/week | 35.6 | 35.6 |  |
| Fruits intake: None | 2.1 | 1.4 | 0.089 |
| ≤3days/week | 32.5 | 33.9 |  |
| >3days/week | 65.4 | 64.7 |  |
| Raw vegetables intake: None | 0.8 | 0.4 | 0.099 |
| ≤3days/week | 18.6 | 19.0 |  |
| >3days/week | 80.6 | 80.6 |  |
| Vigorous physical activity ≥0.5hours/week | 57.6 | 65.6 | <0.001 |
| Central obesity (BMI>30kg/m^2^) | 17.0 | 14.8 | 0.015 |
| Hypertension | 38.5 | 38.3 | 0.966 |
| Area: Basel | 10.9 | 15.5 | <0.001 |
| Wald | 15.4 | 20.4 | <0.001 |
| Davos | 8.7 | 7.2 | 0.016 |
| Lugano | 17.7 | 12.1 | <0.001 |
| Montana | 10.8 | 6.6 | <0.001 |
| Payerne | 12.8 | 16.3 | <0.001 |
| Aarau | 13.9 | 12.5 | 0.063 |
| Geneva | 9.7 | 9.4 | 0.653 |
| Mean (SD) |  |  | T-test |
| Age (years) | 53.3(11.4) | 50.9(11.5) | <0.001 |
| Body mass index (kg/m^2^) | 26.0(4.6) | 25.7(4.4) | 0.005 |
| Blood glucose (mmol/L) | 5.6(1.6) | 5.6(1.5) | 0.996 |
| Triglycerides (mmol/L) | 1.9(1.3) | 1.8(1.2) | <0.001 |
| High-density lipoproteins (mmol/L) | 1.5(0.5) | 1.5(0.4) | 0.7 |
| Neighbourhood SEI | 63.1(10.0) | 63.6(10.4) | 0.030 |
| Pack-years of smoking | 11.0(18.7) | 11.1(18.6) | 0.753 |
| 10-year PM_10_ (µg/m^3^) | 22.5(7.9) | 22.3(6.9) | 0.482 |
| 10-year NO_2_ (µg/m^3^) | 27.4(11.4) | 26.8(10.8) | 0.018 |

ETS: environmental tobacco smoke. VGDF: vapours, gases, dusts and fumes. MVPA: moderate to vigorous physical activity. Hypertension defined as blood pressure >130/85 mm Hg or treatment of previously diagnosed hypertension. SEI: socio-economic index expressed as a percentage. PM_10_: particulate matter <10µm in diameter from all sources. NO_2_: nitrogen dioxide.
