# Supplementary material for: Long-Term Exposure to Ambient Air Pollution and Metabolic Syndrome in Adults
Source: PLoS One. 2015 Jun 23;10(6):e0130337. doi: 10.1371/journal.pone.0130337 (PMC4478007; doi:10.1371/journal.pone.0130337)
Supplement: S2 Table — MetS-W: World Health Organization-defined metabolic syndrome. MetS-I: International Diabetes Federation-defined metabolic syndrome. Model 1: Crude; Model 2: Model 1+ age, sex, educational attainment, neighbourhood socio-economic index, occupational exposure to vapours, gases, dusts or fumes, smoking status, smoked pack-years, exposure to passive smoke, consumption of fruits and raw vegetables, and physical activity; Model 3: Model 2+ body mass index. PM10: particulate matter <10μm in diameter from all sources. NO2: nitrogen dioxide. OR: odds ratio. CI: confidence interval. OR values refer to increments of 10μg/m3 in PM10 and NO2 exposure respectively. Participants’ study area was treated as a random effect in all models. N = 3684 (DOCX) [file pone.0130337.s002.docx]

S2 Table: Incidence rate ratio (IRR) of metabolic syndrome in association with air pollutants.

|  | Model | 10-year mean PM_10_  IRR (95%CI) | 10-year mean NO_2_  IRR (95%CI) |
| --- | --- | --- | --- |
| MetS-W; Cases=382 | Model 1 | 1.55 (1.31, 1.83) | 1.18 (1.02, 1.37) |
|  | Model 2 | 1.45 (1.25, 1.69) | 1.17 (1.02, 1.34) |
|  | Model 3 | 1.52 (1.35, 1.70) | 1.19 (1.02, 1.40) |
| MetS-I ^a^ ; Cases=771 | Model 1 | 1.14 (1.03, 1.26) | 1.07 (1.00, 1.14) |
|  | Model 2 | 1.08 (1.00, 1.18) | 1.03 (0.99, 1.07) |
|  | Model 3 | 1.12 (1.04, 1.19) | 1.06 (1.01, 1.11) |
| MetS-A^b^; Cases=663 | Model 1 | 1.11 (1.02, 1.20) | 1.04 (0.96, 1.11) |
|  | Model 2 | 1.06 (0.98, 1.15) | 1.00 (0.98, 1.02) |
|  | Model 3 | 1.09 (1.00, 1.19) | 1.02 (0.96, 1.08) |

MetS-W: World Health Organization-defined metabolic syndrome. MetS-I: International Diabetes Federation-defined metabolic syndrome. Model 1: Crude; Model 2: Model 1+ age, sex, educational attainment, neighbourhood socio-economic index, occupational exposure to vapours, gases, dusts or fumes, smoking status, smoked pack-years, exposure to passive smoke, consumption of fruits and raw vegetables, and physical activity; Model 3: Model 2+ body mass index. PM_10_: particulate matter <10µm in diameter from all sources. NO_2_: nitrogen dioxide. OR: odds ratio. CI: confidence interval. OR values refer to increments of 10µg/m^3^ in PM_10_ and NO_2_ exposure respectively. Participants’ study area was treated as a random effect in all models. N=3684 ^a^ MetS-I defined using predicted waist circumference and European cut-off for central obesity (≥94cm for men and ≥80cm for women). ^b^ MetS-A defined using predicted waist circumference and North-American cut-off for central obesity (≥102cm for men and ≥88cm for women).
