# Supplementary material for: Long-Term Exposure to Ambient Air Pollution and Metabolic Syndrome in Adults
Source: PLoS One. 2015 Jun 23;10(6):e0130337. doi: 10.1371/journal.pone.0130337 (PMC4478007; doi:10.1371/journal.pone.0130337)
Supplement: S3 Table — MetS-W: World Health Organization-defined metabolic syndrome. MetS-I: International Diabetes Federation-defined metabolic syndrome. Model 1: Crude; Model 2: Model 1+ age, sex, educational attainment, neighbourhood socio-economic index, occupational exposure to vapours, gases, dusts or fumes, smoking status, smoked pack-years, exposure to passive smoke, consumption of fruits and raw vegetables, and physical activity; Model 3: Model 2+ body mass index. PM10: particulate matter <10μm in diameter from all sources. NO2: nitrogen dioxide. OR: odds ratio. CI: confidence interval. OR values refer to increments of 10μg/m3 in PM10 and NO2 exposure respectively. Participants’ study area was treated as a random effect in all models. N = 367 (DOCX) [file pone.0130337.s003.docx]

S3 Table: Association between air pollutants and metabolic syndrome (8 hours fasting time).

|  | Model | 10-year mean PM_10_ OR (95%CI) | 10-year mean NO_2_ OR (95%CI) |
| --- | --- | --- | --- |
| MetS-W  Cases=34 | Model 1 | 1.41 (0.76, 2.61) | 1.17 (0.80, 1.72) |
|  | Model 2 | 1.54 (0.86, 2.76) | 1.11 (0.75, 1.64) |
|  | Model 3 | 1.62 (0.81, 3.26) | 1.16 (0.73, 1.84) |
| MetS-I^a^ Cases=62 | Model 1 | 1.82 (0.91, 3.63) | 1.13 (0.77, 1.64) |
|  | Model 2 | 2.26 (0.93, 5.52) | 1.23 (0.74, 2.05) |
|  | Model 3 | 2.23 (1.04, 4.79) | 1.30 (0.78, 2.19) |
| MetS-A^b^ Cases=56 | Model 1 | 1.35 (0.85, 2.15) | 0.94 (0.71, 1.26) |
|  | Model 2 | 1.51 (0.77, 2.97) | 0.85 (0.57, 1.26) |
|  | Model 3 | 1.56 (0.82, 2.98) | 0.87 (0.78, 1.26) |

MetS-W: World Health Organization-defined metabolic syndrome. MetS-I: International Diabetes Federation-defined metabolic syndrome. Model 1: Crude; Model 2: Model 1+ age, sex, educational attainment, neighbourhood socio-economic index, occupational exposure to vapours, gases, dusts or fumes, smoking status, smoked pack-years, exposure to passive smoke, consumption of fruits and raw vegetables, and physical activity; Model 3: Model 2+ body mass index. PM_10_: particulate matter <10µm in diameter from all sources. NO_2_: nitrogen dioxide. OR: odds ratio. CI: confidence interval. OR values refer to increments of 10µg/m^3^ in PM_10_ and NO_2_ exposure respectively. Participants’ study area was treated as a random effect in all models. N=367 ^a^ MetS-I defined using predicted waist circumference and European cut-off for central obesity (≥94cm for men and ≥80cm for women). ^b^ MetS-A defined using predicted waist circumference and North-American cut-off for central obesity (≥94cm for men and ≥80cm for women).
