# Supplementary material for: Long-Term Exposure to Ambient Air Pollution and Metabolic Syndrome in Adults
Source: PLoS One. 2015 Jun 23;10(6):e0130337. doi: 10.1371/journal.pone.0130337 (PMC4478007; doi:10.1371/journal.pone.0130337)
Supplement: S4 Table — MetS-W: World Health Organization-defined metabolic syndrome. MetS-I: International Diabetes Federation-defined metabolic syndrome. Model 1: Crude; Model 2: Model 1+ age, sex, educational attainment, neighbourhood socio-economic index, occupational exposure to vapours, gases, dusts or fumes, smoking status, smoked pack-years, exposure to passive smoke, consumption of fruits and raw vegetables, and physical activity; Model 3: Model 2+ body mass index. PM10: particulate matter <10μm in diameter from all sources. NO2: nitrogen dioxide. OR: odds ratio. CI: confidence interval. OR values refer to increments of 10μg/m3 in PM10 and NO2 exposure respectively. Participants’ study area was treated as a random effect in all models. N = 3684 (DOCX) [file pone.0130337.s004.docx]

S4 Table: Association between air pollutants and metabolic syndrome (two-pollutant models).

|  | Model | 10-year meanPM_10_ OR (95%CI) | 10-year mean NO_2_ OR (95%CI) |
| --- | --- | --- | --- |
| MetS-W; Cases=382 | Model 1 | 1.79 (1.40, 2.30) | 0.90 (0.72, 1.13) |
|  | Model 2 | 1.65 (1.21, 2.25) | 0.96 (0.76, 1.21) |
|  | Model 3 | 1.84 (1.40, 2.42) | 0.94 (0.78, 1.13) |
| MetS-I ^a^ ; Cases=771 | Model 1 | 1.24 (1.00, 1.54) | 0.99 (0.86, 1.54) |
|  | Model 2 | 1.21 (0.94, 1.57) | 1.00 (0.84, 1.19) |
|  | Model 3 | 1.25 (0.97, 1.61) | 1.04 (0.88, 1.23) |
| MetS-A^b^ ; Cases=663 | Model 1 | 1.25 (1.04, 1.50) | 0.91 (0.80, 1.03) |
|  | Model 2 | 1.26 (1.04, 1.53) | 0.89 (0.77, 1.01) |
|  | Model 3 | 1.28 (1.04, 1.58) | 0.93 (0.81, 1.08) |

MetS-W: World Health Organization-defined metabolic syndrome. MetS-I: International Diabetes Federation-defined metabolic syndrome. Model 1: Crude; Model 2: Model 1+ age, sex, educational attainment, neighbourhood socio-economic index, occupational exposure to vapours, gases, dusts or fumes, smoking status, smoked pack-years, exposure to passive smoke, consumption of fruits and raw vegetables, and physical activity; Model 3: Model 2+ body mass index. PM_10_: particulate matter <10µm in diameter from all sources. NO_2_: nitrogen dioxide. OR: odds ratio. CI: confidence interval. OR values refer to increments of 10µg/m^3^ in PM_10_ and NO_2_ exposure respectively. Participants’ study area was treated as a random effect in all models. N=3684 ^a^ MetS-I defined using predicted waist circumference and European cut-off for central obesity (≥94cm for men and ≥80cm for women). ^b^ MetS-A defined using predicted waist circumference and North-American cut-off for central obesity (≥102cm for men and ≥88cm for women).
