# Supplementary material for: Long-Term Exposure to Ambient Air Pollution and Metabolic Syndrome in Adults
Source: PLoS One. 2015 Jun 23;10(6):e0130337. doi: 10.1371/journal.pone.0130337 (PMC4478007; doi:10.1371/journal.pone.0130337)
Supplement: S5 Table — Fully adjusted models include age, sex, educational attainment, neighbourhood socio-economic index, occupational exposure to vapours, gases, dusts and fumes, smoking status, smoked pack-years, exposure to passive smoke, consumption of fruits and raw vegetables, physical activity and body mass index. NO2: nitrogen dioxide. All analyses were done with four-hour fasting participants. Participants’ study area was treated as a random effect in all models. OR: Odds ratios OR values refer to increments of 10μg/m3 in NO2 exposure. Total N = 3684; N(age≤50) = ; N(males) = 1746; N(physically-active) = 2115; N(never-smoker) = 1623; N(diabetes) = 144. (DOCX) [file pone.0130337.s005.docx]

S5 Table: Effect modification of NO_2_ and metabolic syndrome association.

|  | MetS-W  OR (95%CI) | MetS-I  OR (95%CI) | MetS-A  OR (95%CI) |
| --- | --- | --- | --- |
| Age: Age≤50 | 1.24 (1.04, 1.49) | 1.05 (0.81, 1.36) | 0.83 (0.65, 1.08) |
| Age>50 | 1.34 (0.91, 1.97) | 1.20 (1.07, 1.34) | 1.12 (1.01, 1.23) |
| P-value | 0.664 | 0.091 | 0.021 |
| Sex: Males | 1.27 (1.01, 1.61) | 1.27 (1.08, 1.48) | 1.10 (0.96, 1.25) |
| Females | 1.22 (1.01, 1.48) | 1.08 (0.94, 1.25) | 1.02 (0.89, 1.16) |
| P-value | 0.258 | 0.407 | 0.434 |
| Vigorous physical activity ≥0.5 hrs/wk: Yes | 1.27 (1.08, 1.49) | 1.26 (1.06, 1.48) | 1.08 (0.95, 1.23) |
| No | 1.22 (0.93, 1.62) | 1.04 (0.90, 1.20) | 1.02 (0.87, 1.18) |
| P-value | 0.299 | 0.077 | 0.593 |
| Never-smoker: Yes | 1.17 (0.94, 1.46) | 1.19 (0.98, 1.46) | 1.11 (0.98, 1.27) |
| No | 1.26 (0.97, 1.62) | 1.20 (1.03, 1.36) | 1.00 (0.85, 1.16) |
| P-value | 0.130 | 0.804 | 0.252 |
| Diabetes: Yes | 0.84 (0.58, 1.23) | 0.49 (0.12, 2.03) | 1.56 (0.76, 3.22) |
| No | 1.22 (1.00, 1.50) | 1.17 (1.05, 1.32) | 1.04 (0.94, 1.16) |
| P-value | 0.110 | 0.091 | 0.597 |

Fully adjusted models include age, sex, educational attainment, neighbourhood socio-economic index, occupational exposure to vapours, gases, dusts and fumes, smoking status, smoked pack-years, exposure to passive smoke, consumption of fruits and raw vegetables, physical activity and body mass index. NO_2_: nitrogen dioxide. All analyses were done with four-hour fasting participants. Participants’ study area was treated as a random effect in all models. OR: Odds ratios OR values refer to increments of 10µg/m^3^ in NO_2_ exposure. Total N=3684; N(age≤50)= ; N(males)=1746 ; N(physically-active)=2115 ; N(never-smoker) =1623 ; N(diabetes)=144.
