# Supplementary material for: Long-Term Exposure to Ambient Air Pollution and Metabolic Syndrome in Adults
Source: PLoS One. 2015 Jun 23;10(6):e0130337. doi: 10.1371/journal.pone.0130337 (PMC4478007; doi:10.1371/journal.pone.0130337)
Supplement: S6 Table — MetS-W: World Health Organization-defined metabolic syndrome. MetS-I: International Diabetes Federation-defined metabolic syndrome. MetS-ATP-III: Adult treatment panel III criteria- defined metabolic syndrome. Model 1: Crude; Model 2: Model 1+ age, sex, educational attainment, neighbourhood socio-economic index, occupational exposure to vapours, gases, dusts or fumes, smoking status, smoked pack-years, exposure to passive smoke, consumption of fruits and raw vegetables, and physical activity; Model 3: Model 2+ body mass index. PM10: particulate matter <10μm in diameter from all sources. NO2: nitrogen dioxide. OR: odds ratio. CI: confidence interval. OR values refer to increments of 10μg/m3 in PM10 and NO2 exposure respectively. Participants’ study area was treated as a random effect in all models. N (Four-hour fasting time) = 3684; N (Eight-hour fasting time) = 367. (DOCX) [file pone.0130337.s006.docx]

S6 Table: Association between air pollution and alternative definitions of metabolic syndrome

|  | Model | 10-year mean PM_10_  OR (95%CI) | 10-year mean NO_2_  OR (95%CI) |
| --- | --- | --- | --- |
| MetS-I ^a^ ; Cases = 492 | Model 1 | 1.07 (0.90, 1.26) | 1.04 (0.92, 1.18) |
|  | Model 2 | 1.05 (0.88, 1.25) | 1.04 (0.91,1.18) |
|  | Model 3 | 1.19 (0.97, 1.46) | 1.15 (1.00, 1.33) |
| MetS-I ^b^ Cases = 479 | Model 1 | 1.15 (0.90, 1.47) | 1.08 (0.93, 1.23) |
|  | Model 2 | 1.11 (0.84, 1.48) | 1.03 (0.88, 1.22) |
|  | Model 3 | 1.12 (0.84, 1.48) | 1.05 (0.88, 1.24) |
| MetS-I ^a, c^ ; Cases=50 | Model 1 | 1.61 (0.84, 3.07) | 1.37 (0.93, 2.03) |
|  | Model 2 | 1.63 (0.87, 3.04) | 1.30 (0.88, 1.91) |
|  | Model 3 | 2.24 (1.01, 4.98) | 1.73 (1.05, 2.85) |
| MetS-I ^b, c^ Cases=39 | Model 1 | 1.51 (0.67, 3.38) | 0.93 (0.59, 1.47) |
|  | Model 2 | 1.78 (0.67, 4.73) | 0.94 (0.54, 1.64) |
|  | Model 3 | 1.98 (0.77, 5.08) | 1.02 (0.57, 1.82) |

MetS-W: World Health Organization-defined metabolic syndrome. MetS-I: International Diabetes Federation-defined metabolic syndrome. MetS-ATP-III: Adult treatment panel III criteria- defined metabolic syndrome.. Model 1: Crude; Model 2: Model 1+ age, sex, educational attainment, neighbourhood socio-economic index, occupational exposure to vapours, gases, dusts or fumes, smoking status, smoked pack-years, exposure to passive smoke, consumption of fruits and raw vegetables, and physical activity; Model 3: Model 2+ body mass index. PM_10_: particulate matter <10µm in diameter from all sources. NO_2_: nitrogen dioxide. OR: odds ratio. CI: confidence interval. OR values refer to increments of 10µg/m^3^ in PM_10_ and NO_2_ exposure respectively. Participants’ study area was treated as a random effect in all models. N (Four-hour fasting time)=3684; N (Eight-hour fasting time)=367. ^a^ MetS-I defined using BMI>30kg/m^2^ to define central obesity. ^b^ MetS-I defined using predicted waist circumference and North-American cut-off for central obesity (≥102cm for men and ≥88cm for women). ^c^ Eight-hour fasting time
