# Supplementary material for: Long-Term Exposure to Ambient Air Pollution and Metabolic Syndrome in Adults
Source: PLoS One. 2015 Jun 23;10(6):e0130337. doi: 10.1371/journal.pone.0130337 (PMC4478007; doi:10.1371/journal.pone.0130337)
Supplement: S7 Table — MetS-W: Metabolic syndrome according to World Health Organization. MetS-I: Metabolic syndrome according to International Diabetes Federation. MetS-A: Metabolic syndrome according to Adult Treatment Panel III criteria. ETS: environmental tobacco smoke. VGDF: vapours, gases, dusts and fumes. IFG defined as fasting blood glucose≥6.1mmol/L and/or diagnosis of type2diabetes. High triglycerides defined as fasting triglycerides≥1.7mmol/L or treatment for this condition. Low HDL defined by IDF as < 1.03 mmol/L (males), < 1.29 mmol/L (females), or treatment for this condition, and by WHO as ≤ 0.9 mmol/L (males), ≤ 1.0 mmol/L (females). Hypertension defined by IDF and ATP-III as blood pressure >130/85 mm Hg and by WHO as ≥140/90, or treatment of previously diagnosed hypertension. SEI: socio-economic index. PM10: particulate matter <10μm in diameter from all sources. NO2: nitrogen dioxide. (DOCX) [file pone.0130337.s007.docx]

S7 Table: Participants’characteristics by self-reported physical activity

| Characteristic (%) | Physical activity <0.5 hours/week  (N=1569) | Physical activity ≥0.5 hours/week  (N=2115) | P-value  (Chi^2^) |
| --- | --- | --- | --- |
| Females | 59.6 | 47.4 | <0.001 |
| Education >9 years | 87.8 | 95.7 | <0.001 |
| Never-smokers | 41.7 | 45.8 | 0.015 |
| ETS exposure | 51.9 | 43.1 | <0.001 |
| Occupational exposure to VGDF | 41.4 | 44.0 | 0.116 |
| Alcohol intake: None | 14.3 | 7.2 | <0.001 |
| ≤ once/day | 73.2 | 84.8 |  |
| > once/day | 12.5 | 8.0 |  |
| Citrus fruits intake: None | 10.0 | 7.2 | 0.002 |
| ≤3days/week | 56.3 | 55.9 |  |
| >3days/week | 33.7 | 36.9 |  |
| Fruits intake: None | 2.4 | 1.8 | 0.187 |
| ≤3days/week | 31.8 | 33.2 |  |
| >3days/week | 65.8 | 65.0 |  |
| Raw vegetables intake: None | 1.0 | 0.6 | 0.318 |
| ≤3days/week | 20.6 | 17.1 |  |
| >3days/week | 78.4 | 82.3 |  |
| Low HDL (WHO) | 16.6 | 13.9 | 0.081 |
| Low HDL (IDF) | 30.4 | 22.7 | <0.001 |
| High triglycerides (≥1.7 mmol/L) | 51.7 | 45.0 | <0.001 |
| Impaired fasting glycaemia (IFG; WHO) | 16.8 | 12.6 | <0.001 |
| Impaired fasting glycaemia (IFG; IDF) | 38.2 | 30.1 | <0.001 |
| Hypertension (WHO) | 41.9 | 35.3 | <0.001 |
| Hypertension (IDF) | 53.2 | 47.9 | 0.002 |
| MetS-I ^a^ | 38.0 | 26.5 | <0.001 |
| MetS-W ^b^ | 13.9 | 7.7 | <0.001 |
| MetS-A^c^ | 24.9 | 18.9 | <0.001 |
| Area: Basel | 9.0 | 12.2 | 0.002 |
| Wald | 12.3 | 17.9 | <0.001 |
| Davos | 6.5 | 10.3 | <0.001 |
| Lugano | 32.0 | 7.0 | <0.001 |
| Montana | 7.9 | 13.0 | <0.001 |
| Payerne | 12.3 | 13.1 | 0.510 |
| Aarau | 9.4 | 17.3 | 0.003 |
| Geneva | 10.4 | 9.2 | 0.236 |
| Mean (SD) |  |  | T-test |
| Age (years) | 55.6 (11.1) | 51.7 (11.3) | <0.001 |
| Body mass index (kg/m^2^) | 26.3 (4.8) | 25.6 (4.2) | 0.004 |
| Predicted waist circumference (cm) | 89.6 (13.5) | 88.7 (12.9) | 0.149 |
| Neighborhood SEI | 61.9 (10.3) | 63.4 (9.7) | <0.001 |
| Davos | 6.5 | 10.3 | <0.001 |
| Lugano | 32.0 | 7.0 | <0.001 |
| Montana | 7.9 | 13.0 | <0.001 |

MetS-W: Metabolic syndrome according to World Health Organization. MetS-I: Metabolic syndrome according to International Diabetes Federation. MetS-A: Metabolic syndrome according to Adult Treatment Panel III criteria. ETS: environmental tobacco smoke. VGDF: vapours, gases, dusts and fumes. IFG defined as fasting blood glucose≥6.1mmol/L and/or diagnosis of type2diabetes. High triglycerides defined as fasting triglycerides≥1.7mmol/L or treatment for this condition. Low HDL defined by IDF as < 1.03 mmol/L (males), < 1.29 mmol/L (females), or treatment for this condition, and by WHO as ≤ 0.9 mmol/L (males), ≤ 1.0 mmol/L (females). Hypertension defined by IDF and ATP-III as blood pressure >130/85 mm Hg and by WHO as ≥140/90, or treatment of previously diagnosed hypertension. SEI: socio-economic index. PM_10_: particulate matter <10µm in diameter from all sources. NO_2_: nitrogen dioxide. ^a^defined as central obesity and any two of IFG, hypertension, low HDL and high triglycerides. ^b^defined as IFG and any two of central obesity, hypertension, low HDL and high triglycerides. ^c^defined as any three of five components.
